# Unsupervised identification of cardiometabolic profiles among adolescents: findings from the PARIS birth cohort study

**DOI:** 10.1007/s00431-023-05311-7

**Published:** 2023-11-18

**Authors:** Léa Lefebvre, Thomas Grunemwald, Karima Hamrene, Céline Roda, Isabelle Momas

**Affiliations:** 1 Université Paris Cité, Sorbonne Paris-Nord, INRAe, INSERM, UMR 1153-CRESS, HERA Team, Paris, France; 2 ADEME - Agence de la transition écologique, Angers, France; 3 Centre d’Examens de Santé de la Caisse Primaire d’Assurance Maladie, Paris, France; 4 Faculté de Pharmacie, 4 Avenue de l’Observatoire, 75006 Paris, France; 5 Cellule Cohorte, Mairie de Paris, Direction de la Santé Publique, Paris, France

**Keywords:** Adolescent, Cardiometabolic, Cohort, Profiles

## Abstract

Metabolic syndrome (MetS) is known as a risk factor in cardiometabolic morbidity but there is no consensus on its definition for teenagers. We aimed to identify cardiometabolic health profiles and which parameters best discriminate them. *K*-means partitioning identified cardiometabolic profiles by sex using data on health measurements of 530 adolescents from the PARIS birth cohort. A discriminant analysis was performed. Cardiometabolic risk score and handgrip strength were also measured. Depending on definitions, MetS prevalence ranged from 0.2% to 1.3%. Two profiles were identified for the entire group and by sex: “healthy” and “at cardiometabolic risk.” Weight and waist-to-height ratio or waist circumference explained more than 87% of the variance in the profile differentiation. The “at cardiometabolic risk” profiles included adolescents with overweight, a waist-to-height ratio over 0.5, and prehypertension. They had higher cardiometabolic risk scores and parents who were more likely to be overweight and have cardiometabolic diseases themselves. They also had higher birthweights, earlier adiposity-rebound and puberty ages, and lower relative handgrip strength.

*  Conclusion*: The two profiles identified, based on cardiometabolic health, were associated with early indicators and handgrip strength. Results suggest that the waist-to-height ratio is a useful clinical tool for screening individuals at cardiometabolic risk and who therefore require clinical follow-up.

**Table:** 

**What is Known:** *• Although there is a need for tools to assess cardiometabolic health during adolescence, there is no consensus on the definition of metabolic syndrome for this age group.*	
**What is Knew:** *• The findings suggest that waist-to-height ratio can serve as a simple and valuable clinical tool for screening individuals at cardiometabolic risk who may require clinical monitoring for early prevention of cardiovascular diseases.*

**Supplementary Information:**

The online version contains supplementary material available at 10.1007/s00431-023-05311-7.

## Introduction

Metabolic syndrome (MetS) is the coexistence of abnormalities, including overweight, hypercholesterolemia, hypertriglyceridemia, high blood pressure, and hyperglycaemia, that increase the risk of cardiovascular disease and type 2 diabetes [1]. Indeed, a meta-analysis reported that MetS in the adult population increases by two and 1.5 the risk of developing cardiovascular disease and all-cause mortality, respectively [2]. Therefore, early detection of cardiometabolic risk in adolescence is important for future adult health. Currently, there is no consensus on the definition of MetS among paediatric researchers, particularly for adolescents; many definitions are adapted from adult definitions with sex- and age-specific thresholds. According to Cook et al*.*’s [3] revised definition of the National Cholesterol Education Program Adult Treatment Panel’s (NCEP/ATP III) [4], a diagnosis of MetS is made when at least three parameters are present: waist circumference(WC) ≥ 90th percentile (≥ p90) according to age and sex [5], triglycerides ≥ 1.1 g/L, high-density lipoprotein (HDL) cholesterol ≤ 0.4 g/L, systolic blood pressure (SBP) or diastolic blood pressure (DBP) ≥ p90 according to sex, age, and height [6], and blood glucose ≥ 1.1 g/L. The International Diabetes Federation’s (IDF’s) [7] paediatric definition sets abdominal obesity as a mandatory condition for diagnosis, plus two other parameters: WC ≥ p90 according to age and sex [5], triglycerides ≥ 1.5 g/L, HDL ≤ 0.4 g/L, SBP or DBP ≥ 130/85 mmHg, and blood glucose ≥ 1.0 g/L. Goodman et al*.* [8] adapted the American Heart Association’s (AHA’s) [9] adult definition; the diagnosis is made when at least three parameters are present: WC ≥ p90 according to age and sex [5], triglycerides ≥ 1.1 g/L, HDL ≤ 0.4 g/L, SBP or DBP ≥ p90 according to sex, age, and height [6], and blood glucose ≥ 1.1 g/L. The paediatric definition developed by Jolliffe and Janssen [10] used growth curves to extrapolate the IDF [11] and NCEP/ATP III [4] values for adolescents, considering those with three or more elevated criteria as having MetS. New tools have been developed to assess cardiometabolic health, such as the continuous metabolic syndrome (cMetS) risk score which seems more reliable in predicting young adult risk in late childhood [12] than categorical definitions of MetS. However there is no standard cMetS; a meta-analysis identified 189 different scores [13] and most of them were internally derived z-scores not allowing for comparison across studies, except for the cMetS risk score developed by Stavnsbo et al*.* [14] that uses an international reference population. Finally, there is still no consensus on methods for assessing cardiometabolic health in adolescents. 

In this context, as part of the PARIS birth cohort study, this research aimed to (1) identify cardiometabolic profiles among adolescents using an unsupervised approach and (2) examine the relevance of these profiles by comparing them with regard to growth features, parental cardiometabolic history, cMetS risk score, and handgrip strength.

## Methods

### PARIS birth cohort

The Pollution and Asthma Risk: an Infant Study (PARIS) birth cohort comprised 3840 healthy new-borns recruited between 2003 and 2006 in five Paris maternity hospitals. The follow-up was based on regular questionnaires and health check-ups [15]. The present study involved adolescents who attended the health check-up at 15–16 years of age. The French Ethics Committees approved the PARIS study (permission nos. 031153, 051289, ID-RCB, 2009-A00824-53, and 2009–12-04 MS2). Parents and adolescents gave written informed consent.

### Assessment of cardiometabolic parameters

Anthropometric parameters, blood pressure, heart rate, handgrip strength, and blood samples were collected during the adolescents’ check-ups. Fasting status as well as the time and type of the last meal were recorded. Blood samples were analysed by the central biochemical laboratory.

Body mass and body composition were measured by multi-frequency bioelectrical impedancemetry using a Tanita MC-780MA segmental analyser (Tanita Corp., Tokyo, Japan). Body composition was assessed by the percentage or mass in kilograms (kg) of fat, muscle, and lean mass. Height was measured, to the nearest 0.1 cm, using a mechanical Kern® height metric MSF 200 N (Kern & Sohn GmbH, Balingen, Germany). The body mass index (BMI) was calculated as body mass (kg)/height (m)^2^. The WHO standards were used to calculate BMI z-scores and classify adolescents with underweight (< -2 standard deviation (SD)), with normal weight (≥ -2SD; < 1SD), with overweight (≥ 1SD; < 2SD), or with obesity (≥ 2SD) [16–18]. Waist and hip circumferences were determined, to the nearest 0.1 cm. Heart rate, SBP, and DBP levels were recorded with an Omron HEM-RML31 cuff blood pressure monitor (Omron Healthcare Co. Ltd, Kyoto, Japan). The average of three measurements was calculated. Handgrip strength was assessed, to the nearest kg, using a JAMAR® hydraulic hand dynamometer, model 5030 J1 (Sammons Preston Rolyan, Bolingbrook, Canada); each measurement was repeated three times in each hand, and the relative handgrip strength (kg/kg) of the dominant hand was calculated. Triglycerides (g/L), blood glucose (g/L), total cholesterol (g/L), and HDL (g/L) concentrations were determined by an enzymatic method (GPO-PAP, hexokinase, CHOD-PAP, and a mixture of polyanions and detergents, respectively) using a Roche Cobas® 6000 (c501) analyser. Low-density lipoprotein (LDL) cholesterol levels (g/L) were calculated using the Friedwald formula [19].

### Sociodemographic factors, growth features, and parental cardiometabolic history

At the maternity hospital, data on a baby’s sex, birthweight, and parents’ socioeconomic status (SES) were collected. SES was categorised based on parents’ higher position. Each child’s adiposity rebound age was determined as the rise in the BMI curve. At 15–16 years, puberty data (perceived body and chest development, voice change, age at menarche), parental history of cardiometabolic disease (diabetes, hypercholesterolemia before 50 years of age, stroke before 45, myocardial infarction before 55 for the father and 65 for the mother, and high blood pressure), parental BMI, and SES were collected.

### Statistical analyses

Statistical analyses were performed using Stata® (version SE 17, Stata Corporation, TX, USA) and R (version 4.2.1, R Development Core Team, 2010) software.

We assessed the normality of quantitative variables using the Shapiro–Wilk test and Henry’s graphical method. If required, variables were log-transformed. Comparisons between participants and non-participants and between sexes were performed using the Chi-squared test or Student’s *t*-test.

MetS was determined using the definitions of Cook et al*.* [3], the IDF [7], Goodman et al*.* [8], and Jolliffe and Janssen [10].

The cMetS risk score was based on the calculation model and reference values proposed by Stavnsbo et al*.* [14]. It was constructed from the average of the z-scores for WC, BMI, SBP, DBP, triglycerides, total cholesterol/HDL ratio, LDL, and blood glucose.

Cardiometabolic profiles at 15–16 years old were identified by an unsupervised *k*-means algorithm [20]. The profiles were constructed in the overall population and by sex. To be included in the analysis, adolescents had to have available data on age, height, weight, BMI, WC, hip circumference, waist-to-height ratio, SBP, DBP, LDL, HDL, triglycerides, blood glucose, fat mass, muscle mass, and lean mass. These parameters were standardised. The number of groups was selected using the Calinski-Harabasz index and their relevance. Profiles were compared using the Chi-squared test, Fisher’s exact test, or Student’s *t*-test, and discriminant analysis was used to identify which variables best explained the distribution of individuals between groups.

Profiles were compared in terms of growth features (birthweight and age at adiposity rebound and puberty), parental cardiometabolic history, parental BMI, cMetS risk score and handgrip strength using the Student’s *t*-test and the Chi-squared test or Fisher’s exact test.

A sensitivity analysis was done to assess the effect of fasting status on profile distribution, by comparing the *k*-means analysis conducted among adolescents who fasted for 10 h and those who did not.

## Results

Table 1 presents the baseline characteristics of the adolescents from the PARIS birth cohort, and their families, who were still being followed up at the age of 15–16 years (*n* = 2117). A total of 617 of these adolescents participated in the health check-up for 15–16 year-olds (Fig. S1). Compared with non-participants, participating adolescents’ parents had higher SES and post-secondary education but there was no difference in the geographical origins of parents or their place of residence when their baby was born. Participating adolescents had older mothers but no differences were found regarding whether or not they had been breastfed and with respect to their sex, weight at birth, or exposure to tobacco smoke.

**Table 1:** Baseline characteristics of adolescents from the PARIS birth cohort who participated, or did not participate, in the health check-up at 15–16 years of age (*n* = 2117)

**Baseline characteristics**	**Participants (*n* = 617)**	**Non-participants (*n* = 1500)**	***p*-value**
Male sex, *n* (%)	312 (50.6)	771 (51.4)	0.7
Weight at birth, kg (mean ± SD)	3.4 ± 0.4	3.4 ± 0.4	0.3
Height at birth, cm (mean ± SD)	50.3 ± 1.9	50.1 ± 1.9	0.03
Place of residence			0.8
Paris city, *n* (%)	387 (62.7)	933 (62.2)	
Paris suburbs, *n* (%)	230 (37.3)	567 (37.8)	
Family socioeconomic status ^a^			0.004
Low, *n* (%)	29 (4.7)	125 (8.3)	
Medium, *n* (%)	159 (25.8)	420 (28.0)	
High, *n* (%)	429 (69.5)	955 (63.7)	
Parents’ level of education ^a^			0.004
Primary, *n* (%)	4 (0.7)	45 (3.0)	
Secondary, *n* (%)	121 (19.6)	309 (20.6)	
Post-secondary, *n* (%)	492 (79.7)	1,146 (76.4)	
Geographical origin of parents			0.2
Two parents born in France, *n* (%)	458 (74.2)	1,070 (71.3)	
At least one parent born outside France, *n* (%)	159 (25.8)	430 (28.7)	
Mother’s age at birth, years (mean ± SD)	33.2 ± 3.9	32.7 ± 4.0	0.008
Breastfed at birth, *n* (%)	508 (83.3)	1,190 (80.1)	0.1
Mother actively smoked during pregnancy, n (%)	55 (8.9)	145 (9.7)	0.6
Smokers at home at birth, *n* (%)	112 (18.4)	299 (20.2)	0.4

*p-*value from Chi-squared/Fisher’s exact test or Student’s *t*-test

*SD* standard deviation

^a^Highest among parents

Descriptions of cardiometabolic health parameters in the whole, male, and female populations are shown in Table 2. Adolescents were, on average, 15.9 (± 0.3) years old at the time of the check-up. Compared to females, males were taller and heavier, and had a higher WC but a lower BMI and hip circumference. Males also had a lower percentage of body fat mass and a higher percentage of lean body mass and muscle mass. The SBP was higher in males while the DBP and heart rate were higher in females. Males had lower LDL, HDL, and blood glucose than females.

**Table 2:** Description and sex comparison of anthropometric and biological parameters of adolescents from the PARIS birth cohort at 15–16 years of age

	**Total, *n* = 617**			**Male, *n* = 312**		**Female, *n* = 305**		
	*n*	Mean ± SD	Min–Max	Mean ± SD	Min–Max	Mean ± SD	Min–Max	*p-*value
Age (years)	617	15.9 ± 0.3	15.3–17.7	15.9 ± 0.3	15.4–17.7	15.9 ± 0.3	15.3–17.4	0.6
Height (cm)	617	171.2 ± 8.4	148.0–195.0	176.3 ± 6.8	154.0–195.0	165.9 ± 6.5	148.0–187.0	< 0.001
Weight (kg)	617	59.2 ± 10.4	33.4–123.9	61.1 ± 10.1	33.4–106.4	57.2 ± 10.4	38.4–123.9	< 0.001
BMI (kg/m^2^)	617	20.2 ± 3.2	13.4–41.4	19.6 ± 2.7	13.4–34.0	20.7 ± 3.5	15.2–41.4	< 0.001
Waist circumference (cm)	611	72.7 ± 8.1	54.0–115.0	73.6 ± 7.5	57.0–107.0	71.8 ± 8.7	54.0–115.0	0.003
Hip circumference (cm)	610	92.2 ± 7.8	60.0–135.0	90.6 ± 7.3	60.0–125.0	93.9 ± 7.9	75.0–135.0	< 0.001
Waist-to-height ratio	611	0.4 ± 0.1	0.3–0.7	0.4 ± 0.1	0.3–0.6	0.4 ± 0.1	0.3–0.7	< 0.001
Body fat mass (%)	545	20.9 ± 7.2	8.7–55.4	15.5 ± 4.3	8.7–36.9	26.1 ± 5.4	17.5–55.4	< 0.001
Lean body mass (%)	545	79.1 ± 7.2	44.6–92.7	84.5 ± 4.3	63.1–92.7	73.8 ± 5.3	44.6–82.6	< 0.001
Muscle mass (%)	545	75.1 ± 6.8	42.3–88.0	80.2 ± 4.0	60.0–88.0	70.1 ± 5.0	42.3–78.3	< 0.001
SBP (mmHg)	617	110.4 ± 9.5	84.0–140.3	113.4 ± 9.3	91.0–140.3	107.4 ± 8.7	84.0–139.0	< 0.001
DBP (mmHg)	617	65.9 ± 6.1	49.0–87.0	64.9 ± 6.3	49.0–86.3	66.8 ± 5.9	52.0–87.0	< 0.001
Heart rate (pulse/minute)	613	67.6 ± 10.7	39.0–116.0	65.4 ± 10.0	39.0–109.0	69.8 ± 11.0	45.0–116.0	< 0.001
LDL (g/L)	608	0.8 ± 0.2	0.2–1.8	0.8 ± 0.2	0.2–1.5	0.9 ± 0.3	0.3–1.8	< 0.001
HDL (g/L)	610	0.6 ± 0.1	0.3–1.0	0.5 ± 0.1	0.3–1.0	0.6 ± 0.1	0.3–0.9	< 0.001
Triglycerides (g/L)	610	0.8 ± 0.4	0.3–4.1	0.8 ± 0.4	0.3–4.1	0.8 ± 0.4	0.3–3.8	0.09
Blood glucose (g/L)	610	0.9 ± 0.1	0.5–2.1	0.9 ± 0.1	0.5–2.1	0.9 ± 0.1	0.6–1.4	0.002

*p-*value from Student’s *t*-test between sexes, *n* varies due to missing data

*BMI* body mass index (weight (kg)/height (m)^2^), *DBP* diastolic blood pressure, *HDL* high-density lipoprotein, *IOTF* International Obesity Task Force, *LDL* low-density lipoprotein, *Max* maximum, *Min* minimum, *SBP* systolic blood pressure, *SD* standard deviation, *WHO* World Health Organization

MetS prevalence was 0.2%, 0.5%, 0.7%, and 1.3%, according to the IDF, Cook et al*.*, Goodman et al*.*, and Jolliffe and Janssen definitions, respectively.

**Fig. 1:**
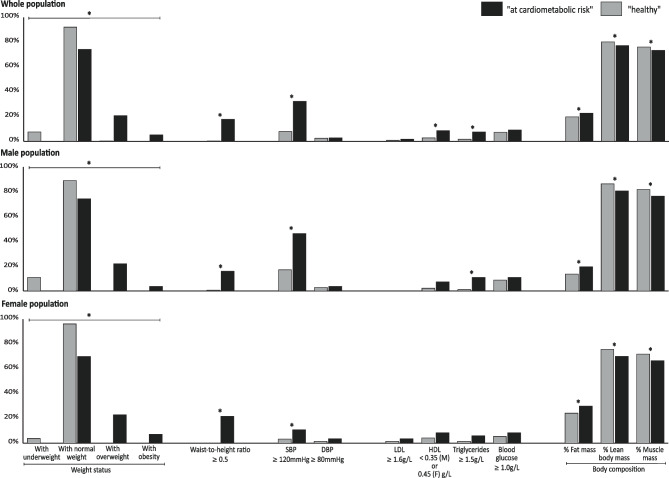
Comparison of anthropometric and biological parameters by identified cardiometabolic health profiles of PARIS birth cohort adolescents at 15–16 years old. DBP, diastolic blood pressure; HDL, high-density lipoprotein; LDL, low-density lipoprotein; SBP, systolic blood pressure. *p-*value < 0.05 is illustrated by asterisk (*) from the Chi-squared test, Fisher’s exact test, and Student’s *t*-test. Not all parameters are here presented, mainly those discretised using established thresholds: weight status [16–18]; waist-to-height ratio [46]; SBP and DBP [6]; LDL, HDL, and triglycerides (medical biology laboratory value thresholds); and blood glucose [47]

A total of 530 adolescents were included in the cluster analysis. Two cardiometabolic profiles were identified in the whole population (*n* = 530) and in males (*n* = 263) and females (*n* = 267) (Fig. 1). The two identified profiles— “healthy” and “at cardiometabolic risk”—showed similar pattern for the whole population and sub-populations. The sensitivity analysis revealed that the classification of adolescents remained unchanged whether or not their profiles were created based on their fasting status. Moreover, no variation in the fasting status was observed between the “at cardiometabolic risk” and “healthy” profiles, regardless of the population. Compared to the “healthy” profiles, the “at cardiometabolic risk” profiles were characterised by a significantly higher weight, height, BMI, waist and hip circumference, and body fat mass, and a lower muscle and lean mass. The “at cardiometabolic risk” profile included all the participants with obesity and overweight, and a higher proportion of those with a waist-to-height ratio > 0.5 and prehypertension. In the whole population, a higher proportion of participants in the “at cardiometabolic risk” profile had low HDL. In the whole and male populations, the “at cardiometabolic risk” profiles included a higher proportion of participants with hypertriglyceridemia. Weight was the most discriminating variable, explaining 77% to 81% of the group differences. For the overall and female populations, WC was the second most discriminating variable, adding 8% and 6%, respectively, to the explanatory power. In the male population, waist-to-height ratio was the second most discriminating variable, adding 12% to the explanatory power.

The mean cMetS risk score of participating adolescents was − 0.12 (± 0.5). In all populations, those in the “at cardiometabolic risk” profiles had a significantly higher cMetS risk score than those in the “healthy” profiles (Table 3). In addition, their parents were more likely to live with obesity and overweight and have cardiometabolic diseases. Those in the “at cardiometabolic risk” profile had a higher birthweight and an earlier adiposity rebound, puberty, and menarche age. Finally, relative handgrip strength was significantly lower among those in the “at cardiometabolic risk” profiles.

**Table 3:** Comparison of associated factors between cardiometabolic profiles of adolescents from the PARIS birth cohort at 15–16 years of age

	**Whole population (*n* = 530)**			**Male (*n* = 263)**			**Female (*n* = 267)**		
	Healthy	Cardiometabolic risk	*p-*value	Healthy	Cardiometabolic risk	*p-*value	Healthy	Cardiometabolic risk	*p-*value
	356 (67.2%)	174 (32.8%)		181 (68.8%)	82 (31.2%)		184 (68.9%)	83 (31.1%)	
Cardiometabolic risk score, mean ± SD	− 0.3 ± 0.4	0.2 ± 0.5	< 0.001	− 0.3 ± 0.3	0.2 ± 0.6	< 0.001	− 0.2 ± 0.4	0.2 ± 0.4	0.002
Perceived body development			< 0.001			< 0.001			< 0.001
Early, *n* (%)	26 (7.9)	43 (26.5)		18 (10.6)	20 (26.7)		13 (7.6)	18 (23.7)	
Same as the others, *n* (%)	241 (73.0)	104 (64.2)		113 (66.9)	50 (66.7)		129 (75.0)	53 (69.7)	
Late, *n* (%)	63 (19.1)	15 (9.3)		38 (22.5)	5 (6.6)		30 (17.4)	5 (6.6)	
Chest development / voice change			0.004			0.8			0.008
Not yet / beginning, *n* (%)	8 (2.5)	1 (0.7)		5 (3.0)	1 (1.4)		3 (1.9)	0	
Already grown / changed, *n* (%)	37 (11.7)	6 (3.8)		14 (8.4)	5 (6.8)		22 (13.8)	2 (2.7)	
Finished, *n* (%)	272 (85.8)	150 (95.5)		148 (88.6)	68 (91.8)		134 (84.3)	72 (97.3)	
Age at menarche									< 0.001
< 12 years old							23 (13.7)	27 (35.5)	
Between 12 and 14 years old							140 (83.3)	48 (63.2)	
> 14 years old							5 (3.0)	1 (1.3)	
Adiposity rebound			0.02			0.04			0.1
Early age, *n* (%)	133 (40.4)	85 (51.2)		59 (34.5)	39 (50.6)		77 (45.8)	43 (54.4)	
Medium age, *n* (%)	156 (47.4)	71 (42.8)		93 (54.4)	34 (44.2)		68 (40.5)	32 (40.5)	
Late age, *n* (%)	40 (12.2)	10 (6.0)		19 (11.1)	4 (5.2)		23 (13.7)	4 (5.1)	
Birthweight, kg, mean ± SD	3.4 ± 0.4	3.6 ± 0.4	< 0.001	3.5 ± 0.4	3.6 ± 0.5	0.02	3.3 ± 0.4	3.5 ± 0.4	< 0.001
Parental history of cardiometabolic disease, *n* (%)^a^	89 (25.7)	63 (37.3)	0.007	43 (24.4)	28 (35.0)	0.08	49 (28.8)	31 (40.3)	0.09
Father’s weight status			< 0.001			0.01			0.01
With underweight, *n* (%)	3 (1.0)	0		2 (1.4)	0		1 (0.6)	0	
With normal weight, *n* (%)	166 (55.9)	43 (31.9)		78 (52.7)	22 (33.3)		87 (55.5)	21 (35.0)	
With overweight/obesity, *n* (%)	128 (43.1)	92 (68.1)		68 (45.9)	44 (66.7)		69 (43.9)	39 (65.0)	
Mother’s weight status			< 0.001			0.005			0.001
With underweight, *n* (%)	22 (6.4)	1 (0.6)		16 (9.4)	0		7 (3.9)	0	
With normal weight, *n* (%)	250 (73.1)	104 (63.8)		117 (68.4)	53 (68.8)		136 (76.4)	47 (60.3)	
With overweight/obesity, *n* (%)	70 (20.5)	58 (35.6)		38 (22.2)	24 (31.2)		35 (19.7)	31 (39.7)	
Relative handgrip strength force, kg/kg, mean ± SD	0.5 ± 0.1	0.5 ± 0.1	< 0.001	0.6 ± 0.1	0.5 ± 0.1	0.004	0.5 ± 0.1	0.4 ± 0.1	< 0.001

*p-*value from Student’s *t*-test, Chi-squared or Fisher’s exact tests to compare profiles

^a^Diabetes, hypercholesterolemia before 50 years old, stroke before 45, myocardial infarction before 55 for the father and 65 for the mother, and high blood pressure

## Discussion

### Key results

To our knowledge, this is the first study to describe MetS in an urban population of adolescents in France. The prevalence of MetS in this Parisian population varies from 0.2% to 1.3% depending on the definition considered. Using unsupervised *k*-means classification, two different groups were identified based on their cardiometabolic health status. The profiles for these two groups differed in terms of their cMetS risk score and were associated with known early determinants of cardiometabolic health as well as handgrip strength, a predictor of overall health.

### Strengths and limitations

This study has several strengths, notably the use of an unsupervised approach (without any a priori assumptions), which, has not been previously applied in this context. This enabled the identification of several cardiometabolic profiles based on well characterised anthropometric, clinical, and biological data. Moreover, health data were collected during a standardised medical examination performed by the same medical team, and biological assays were performed in a single laboratory, thereby reducing measurement and classification bias. This study benefited from the prospective data collected throughout the follow-up of the cohort (birthweight, adiposity rebound age, parental cardiometabolic history, and parental BMI).

This study has also limitations. Approximately one-third of adolescents still followed in the PARIS birth cohort attended the health check-up at 15–16 year-olds. This was primarily due to logistical constraints (half a weekday check-up, COVID-19 lockdown…). As often observed in cohort follow-up involving health examination, participating adolescents were from families with a higher SES than non-participants; and SES is a well-known risk factor for cardiometabolic health [21]. Finally, fasting status varied among adolescents, health check-ups taking part either in the morning or in the afternoon, but sensitivity analysis showed that it had no impact on profile determination.

### Cardiometabolic health in adolescents from the PARIS birth cohort

This study documented a lower prevalence of MetS in adolescents (0.3–1.2%) compared to previous European (1.4–5.8%) [22, 23] and American (National Health and Nutrition Examination Survey (NHANES), 6.8%) studies [24]. The population in this current study seems to be healthier, possibly due to the inclusion of adolescents from higher SES, a factor known to have an impact on adolescents’ cardiometabolic health [21].

MetS definitions enable to diagnose patients with established cardiometabolic health issues, which are uncommon among adolescents. However, teenagers can present early weak signals of cardiometabolic risk that MetS definitions are not suitable to detect. Therefore, a tool to identify at-risk but non-pathological individuals during the transition period of adolescence could be useful.

Several authors have used a cMetS to avoid potential misclassification [25–28]. According to Fernandez-Aparicio [29], cardiometabolic risk score based on the z-score is an accurate and efficient method that can be used to determine MetS risk in adolescents. Indeed, a recent meta-analysis [30] reported a pooled sensitivity and a specificity of cMetS risk scores in predicting the risk of MetS: 0.90 (95% confidence interval (CI), 0.83–0.95) and 0.86 (95% CI, 0.83–0.89), respectively. The negative and positive likelihood ratios (0.11 (95% CI, 0.0063–0.21) and 6.5 (95% CI, 5.0–8.6), respectively) indicated the ability of cMetS risk scores to separate healthy and at-risk individuals. Kelly et al*.* [12] used cMetS risk score and MetS in 13 and 22 year-olds and showed cMetS risk score in adolescence was more predictive of adult cardiometabolic health than MetS. Nevertheless, most scores were centred on the sample mean [31–33], making comparisons between studies impossible. Stavnsbo et al*.* [27] proposed a unified approach with international age- and sex-specific reference values to calculate cMetS risk score. Based on this approach, Parisian adolescents had a lower risk score than the international reference population (− 0.12 ± 0.5), which confirms the healthier status of the adolescents in this current study.

### Cardiometabolic profiles in adolescents from the PARIS birth cohort

The *k*-means approach classified adolescent into two groups according to their cardiometabolic health. One group was more at risk of cardiometabolic disease than the other, characterised by a higher cardiometabolic risk score than the international reference (0.2 ± 0.5); the other group, considered “healthy,” had a much lower score (− 0.3 ± 0.4).

The profiles were mainly discriminated by weight and WC or waist-to-height ratio, both of which explained more than 87% of the variance. Almost all adolescents with a waist-to-height ratio above 0.5 were in the at-risk profiles, as well as all adolescents who lived with overweight and obesity. These results are consistent with a meta-analysis reporting that these anthropometric parameters were the best screening tools for paediatric MetS [34]. Another meta-analysis, showed that a high waist-to-height ratio (> 0.5) doubled the risk of having two or more MetS criteria after adjustment for BMI [35].

These profiles seem relevant with regard to known determinants of cardiometabolic health: birthweight, adiposity rebound, puberty, parental BMI, and cardiometabolic diseases.

Adolescents in the “at cardiometabolic risk” profiles had higher birthweight than those in the “healthy” profiles. Studies showed that increasing birthweight was associated with increasing trends of prevalence of high WC [36] and risk of having overweight [37]. Tam et al. [38] found that both low and high birthweights were associated with an increased cardiometabolic risk, supporting the relationship observed in this study. As the population for this current study was composed of full-term new-borns, low birthweight was too rare to be examined.

The age of adiposity rebound was lower in the at-risk group for the whole population and in males, and it tended to be lower in females. A birth cohort study in Porto showed that children with very early or early adiposity rebound had higher cardiometabolic parameters, such as BMI, WC, SBP, and triglycerides [39].

Adolescents with early puberty were more likely to be at-risk, and females in this profile experienced earlier menarche. Puberty affects body composition in adolescence [40], and its early onset is linked to overweight and obesity in females [41]. Early menarche is associated with a higher prevalence of MetS in young females [42].

The identified profiles were associated with both cardiometabolic history and weight status of the participants’ parents. A meta-analysis found that parental weight status is positively associated with child weight status (pooled odds ratio (OR):2.22;95%CI:2.09–2.36) [43].

Cardiometabolic profiles were associated with a marker of overall health, and adolescents in the at-risk profiles had a lower relative handgrip strength than those in the healthy profiles. Kim et al*.* [44] found a negative association between relative handgrip strength and cardiometabolic risk in adolescents. Ramirez-Velez et al*.* [45] showed that relative handgrip strength can be used to screen adolescents with high cardiometabolic risk.

The waist-to-height ratio and WC are useful and easy to use clinical tools to detect children potentially at risk of cardiometabolic pathologies and for whom a clinical follow-up is needed. Follow-up of the PARIS birth cohort will enable researchers to study the cardiometabolic outcomes of these adolescents.

## Conclusion

Unsupervised classification allowed the identification of two different groups, in the total population and by sex, based on their cardiometabolic health. These profiles were associated with early signals (birthweight, age of adiposity rebound, puberty), and parents’ BMI and cardiometabolic diseases. Relative handgrip strength, a predictor of health, and cMetS risk score were associated with these groups. Although the cardiometabolic health of Parisian adolescents seems to be good, a group was identified in which cardiometabolic risk appeared to be higher. These results show that it is essential to monitor cardiometabolic health from an early age in order to follow those who are most at risk and subsequently initiate treatment to prevent adult disorders.

### Supplementary Information

Below is the link to the electronic supplementary material.

Supplementary file1 (PDF 96 KB)

## Data Availability

The data supporting this research is available upon reasonable request.

## Abbreviations

AHA: American Heart Association
BMI: Body mass index
cMetS: Continuous metabolic syndrome
DBP: Diastolic blood pressure
HDL: High-density lipoprotein
IDF: International Diabetes Federation
LDL: Low-density lipoprotein
MetS: Metabolic syndrome
NCEP/ATPIII: National Cholesterol Education Program Adult Treatment Panel
NHANES: National Health and Nutrition Examination Survey
OR: Odd ratio
PARIS: Pollution and asthma risk: an infant study
SBP: Systolic blood pressure
SD: Standard deviation
SES: Socioeconomic status
WC: Waist circumference
WHO: World Health Organization
