# Supplementary figures and images for: Unsupervised identification of cardiometabolic profiles among adolescents: findings from the PARIS birth cohort study
Source: Eur J Pediatr. 2023 Nov 18;183(2):715–25. doi: 10.1007/s00431-023-05311-7 (PMC10912260; doi:10.1007/s00431-023-05311-7)

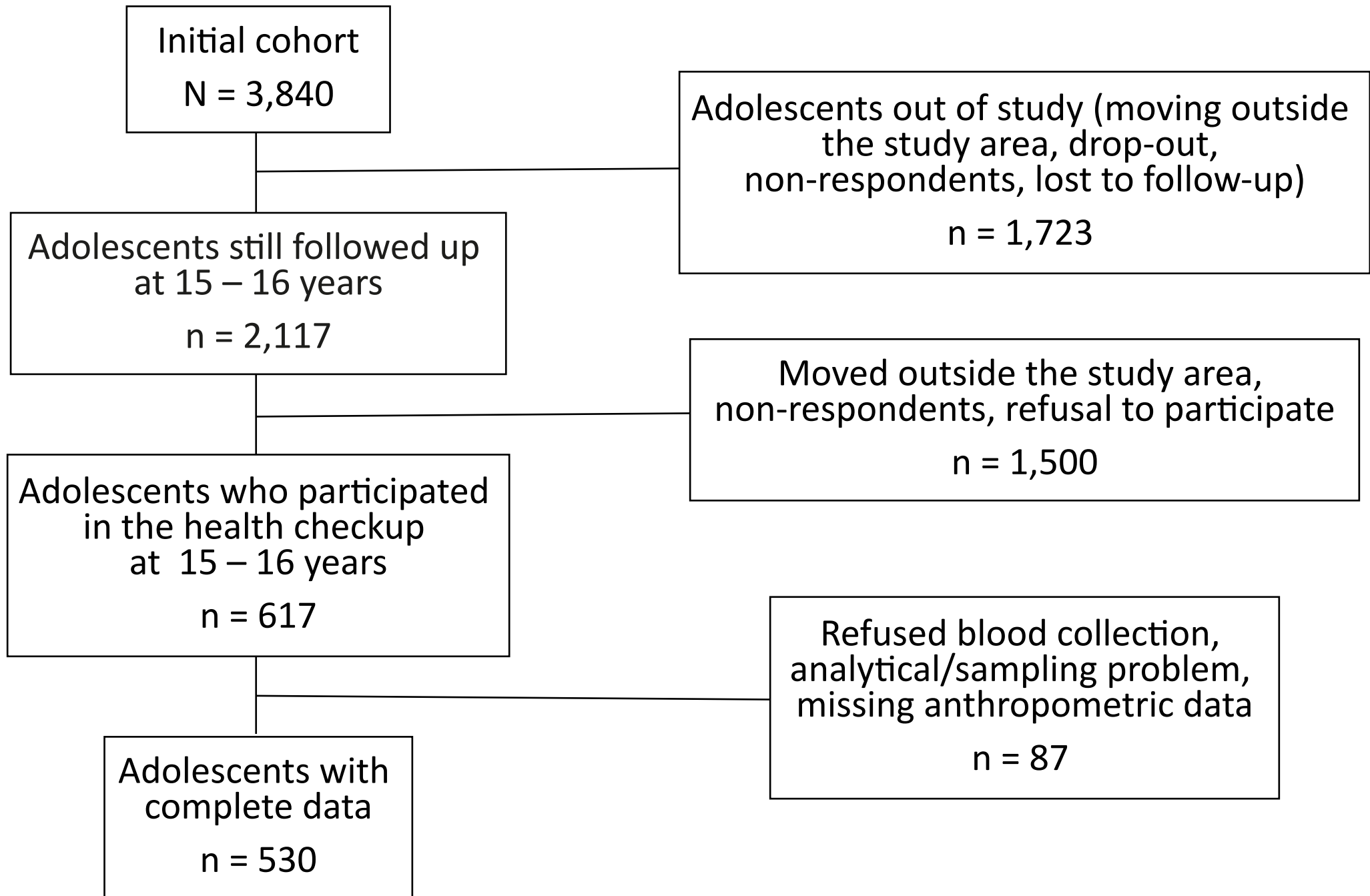

Supplement: Supplementary file 1 — Supplementary file1 (PDF 96 KB) [file 431_2023_5311_MOESM1_ESM.pdf]
